# Supplementary material for: Anthropogenic combustion iron as a complex climate forcer
Source: Nat Commun. 2018 Apr 23;9:1593. doi: 10.1038/s41467-018-03997-0 (PMC5913250; doi:10.1038/s41467-018-03997-0)
Supplement: Supplementary file 1 — Supplementary Information [file 41467_2018_3997_MOESM1_ESM.pdf]

Supplementary Information

Anthropogenic combustion iron as a complex climate forcer

Matsui et al.

Supplementary Figures

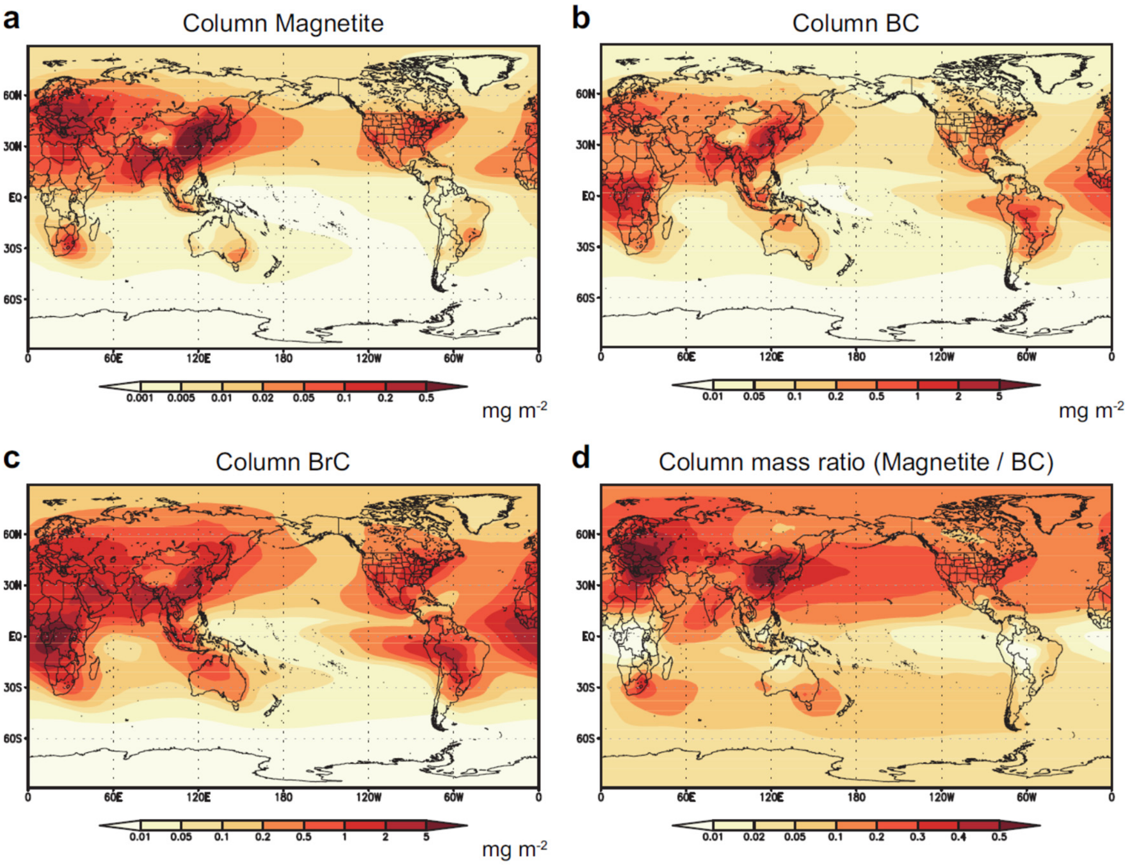

Supplementary Figure 1. Global distributions of column aerosol mass concentrations. a-c, (a) Magnetite, (b) black carbon (BC), and (c) brown carbon (BrC) concentrations in the NEW simulation (5-year mean). **d,** The ratio between magnetite and BC concentrations in the NEW simulation (5-year mean).

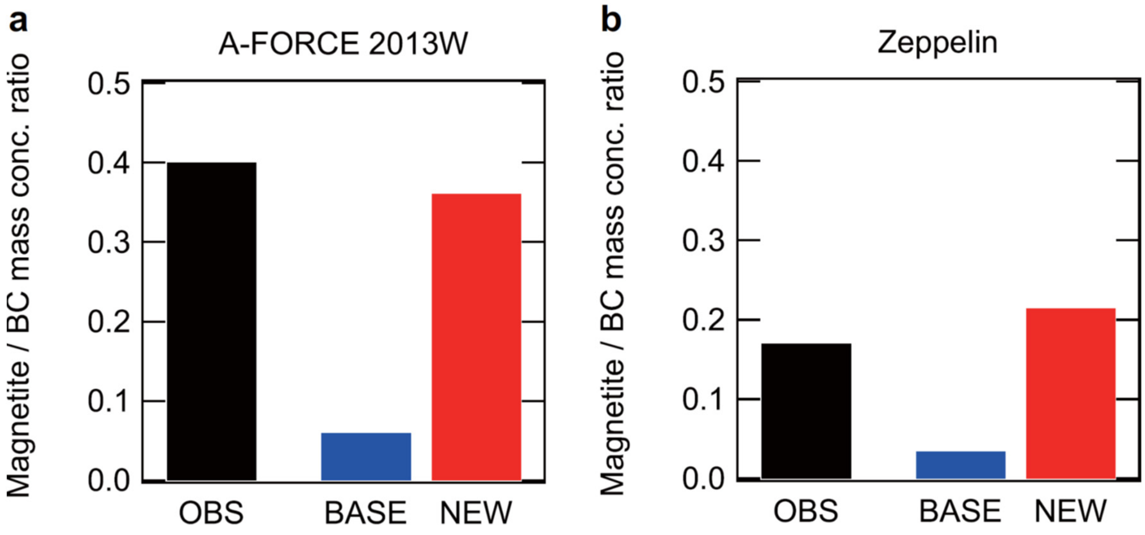

Supplementary Figure 2. Comparisons between observed and simulated magnetite/BC ratios. a-b, Mean magnetite/BC mass ratio for observation (OBS) and model simulations (BASE and NEW) (a) during the A-FORCE 2013W aircraft campaign (Feb-Mar 2013) and (b) intensive surface measurements at Zeppelin (Mar 2017).

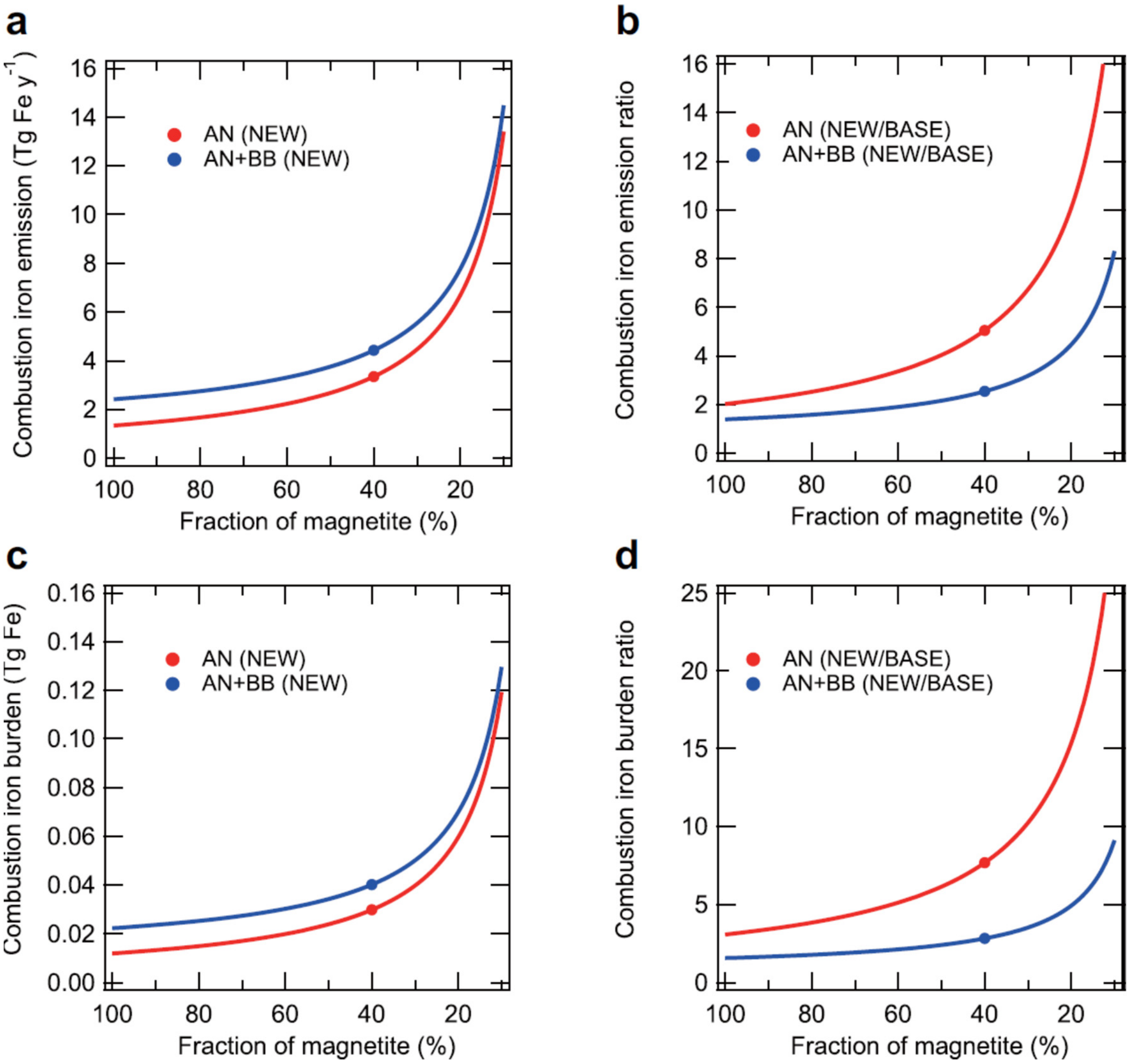

Supplementary Figure 3. Sensitivity of combustion iron estimates to the assumption of magnetite fraction. **a,** The emission flux of combustion iron. **b,** The ratio of combustion iron emission flux between the NEW and BASE simulations. **c,** The atmospheric burden of combustion iron. **d,** The ratio of combustion iron burden between the NEW and BASE simulations.

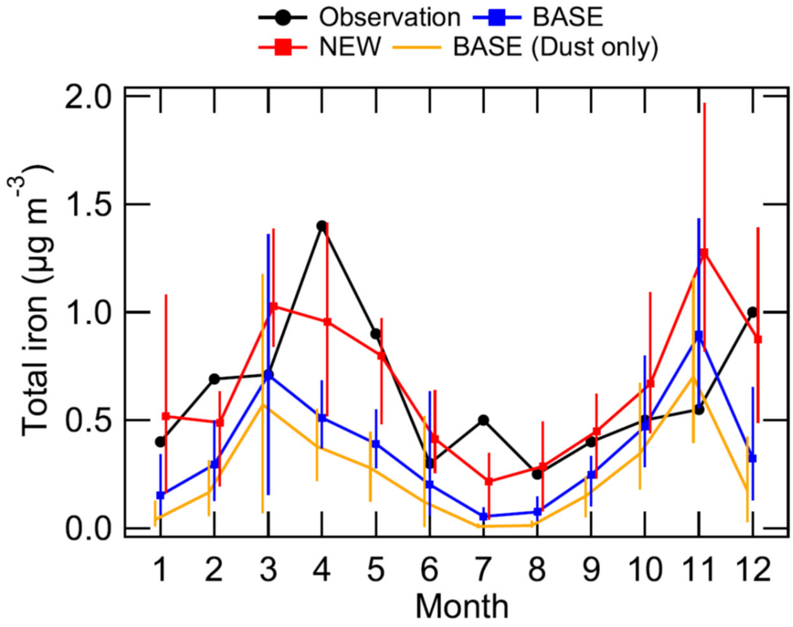

Supplementary Figure 4. Monthly variations of total iron concentrations at Gosan (126.17E, 33.28N), East Asia. Observed total iron data (black) from Fig. 6 of Luo et al.[1]. Simulated total iron results are shown for the BASE (blue) and NEW (red) simulations (5-year mean). Iron concentrations from dust sources only is shown for the BASE simulation (orange, 5-year mean). Vertical bars show the interannual variability (maximum-minimum range) of simulated results.

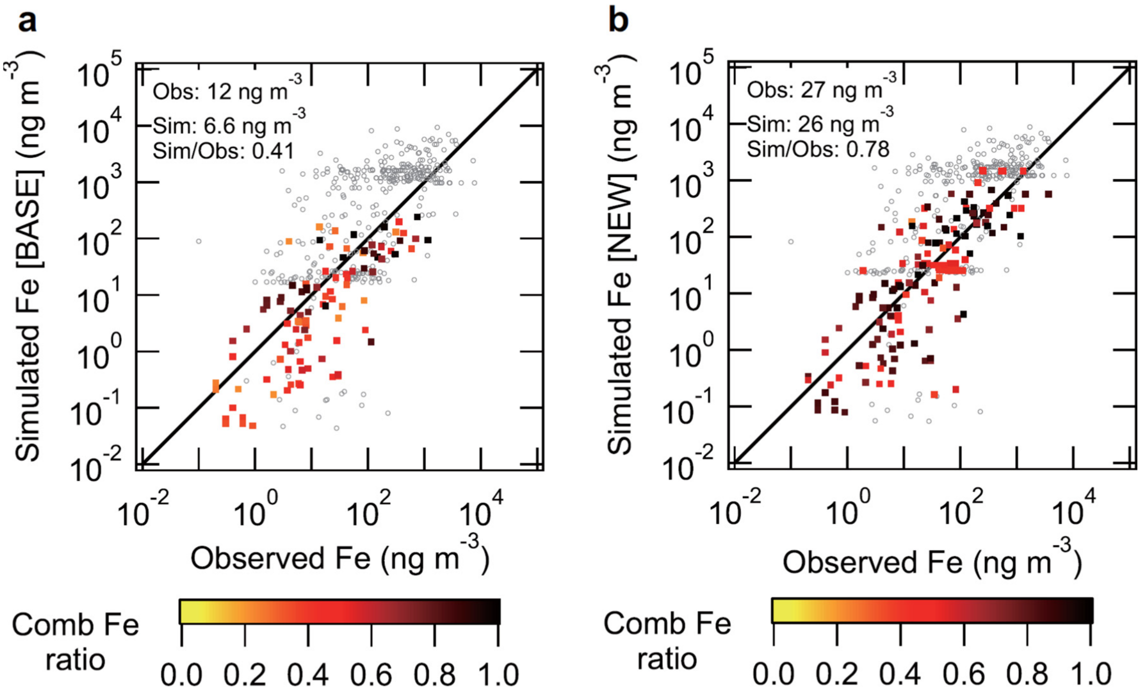

Supplementary Figure 5. Scatterplots of observed and simulated total iron concentrations. a-b, Comparisons between observed and simulated total iron concentrations at the surface for the (a) BASE and (b) NEW simulations (5-year mean). Colored squares are data which have large contributions from combustion sources (> 20% of total in simulations: the number of data points is 99 in **a** and 149 in **b**). Gray open circles are dust-dominant data (the contribution of combustion sources is less than 20% in simulations). Median values of colored squares are shown for observations (Obs), simulations (Sim), and the ratio of simulations to observations (Sim/Obs) in **a-b.**

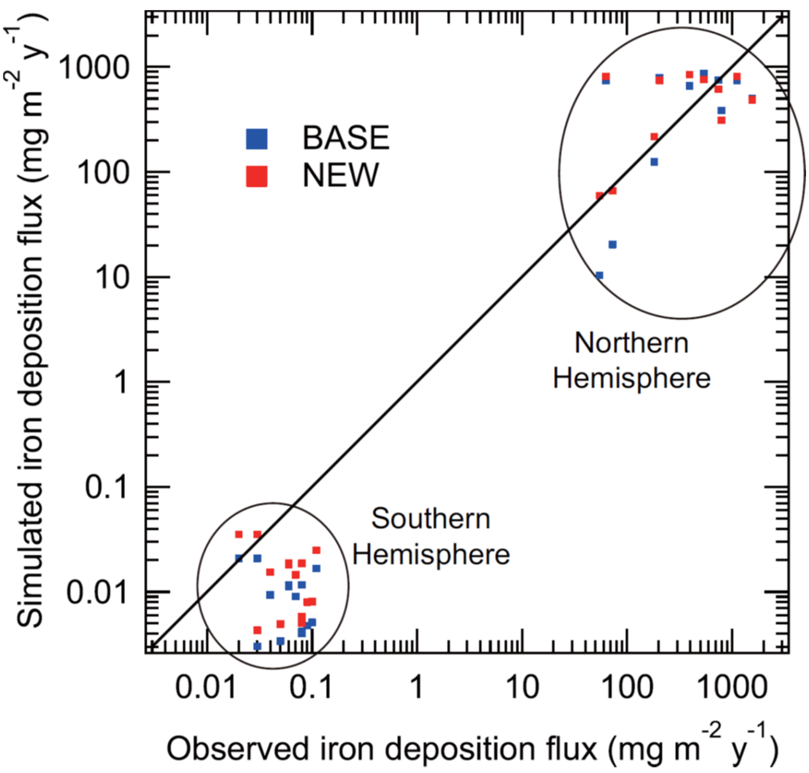

Supplementary Figure 6. Atmospheric deposition flux of total iron to the ocean. Observed and simulated total iron deposition flux for the BASE (blue) and NEW (red) simulations. Observed data are from Mahowald et al.[2].

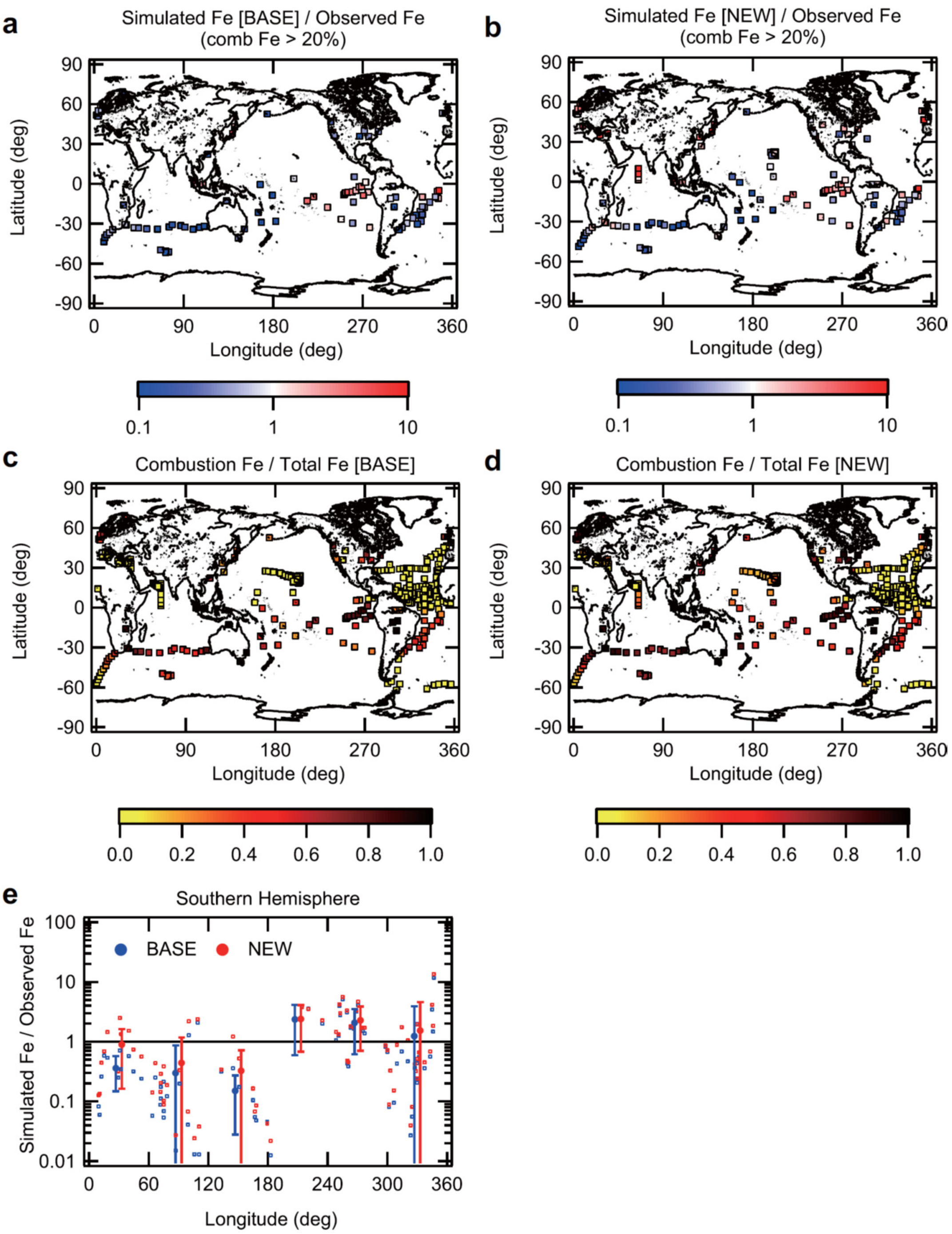

Supplementary Figure 7. Ratios of iron concentrations at the surface. a-b, The ratio between simulated and observed total iron concentrations for the (a) BASE and (b) NEW simulations for data which have large contributions from combustion sources (> 20% of total in simulations) (5-year mean). **c-d,** The ratio of combustion iron to total iron concentrations for the (c) BASE and (d) NEW simulations (5-year mean). **e**, The ratio between simulated and observed total iron concentrations in the Southern Hemisphere (5-year mean). Figures were made using IGOR Pro, WaveMetrics Inc., Oswego, OR.

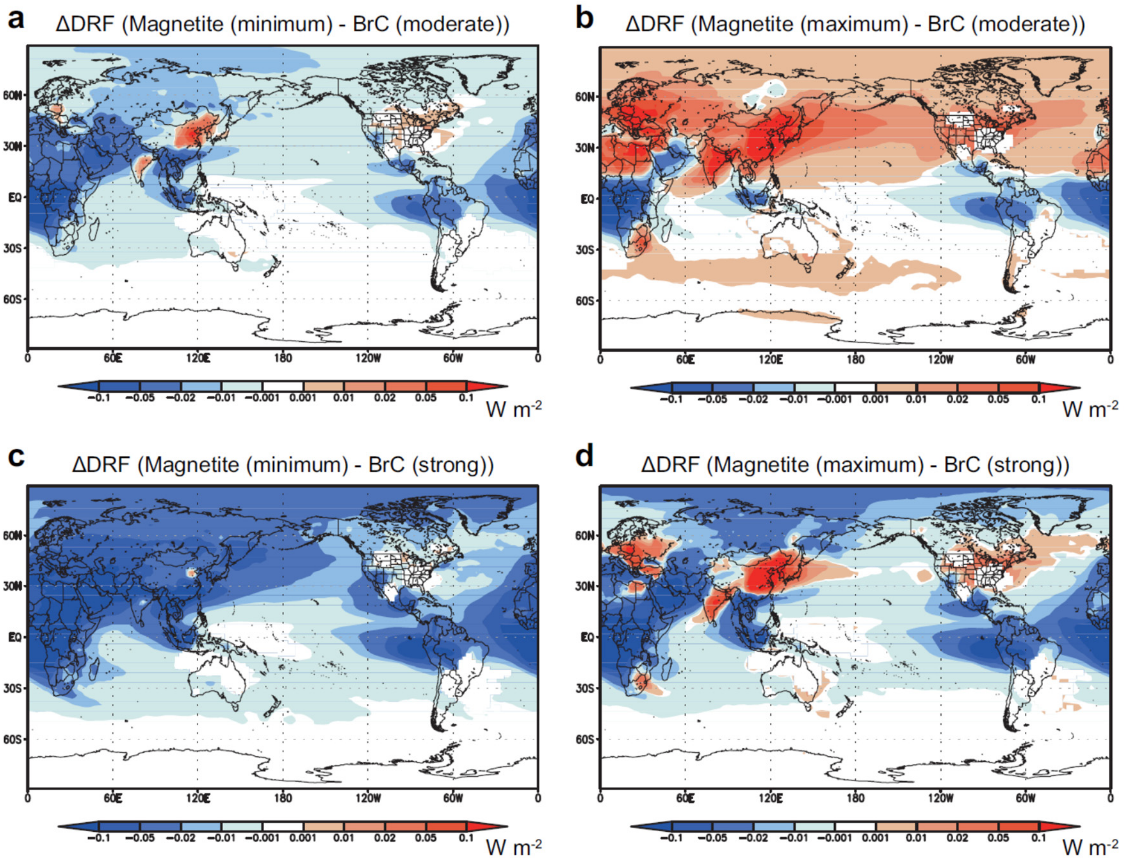

Supplementary Figure 8. Direct radiative forcing differences between magnetite and brown carbon. a-d, Differences of direct radiative forcing (DRF) for 4 (2 × 2) cases (global and 5-year mean): 2 magnetite DRF (minimum and maximum estimates) and 2 brown carbon (BrC) DRF (strong and moderate absorption) (see Methods).

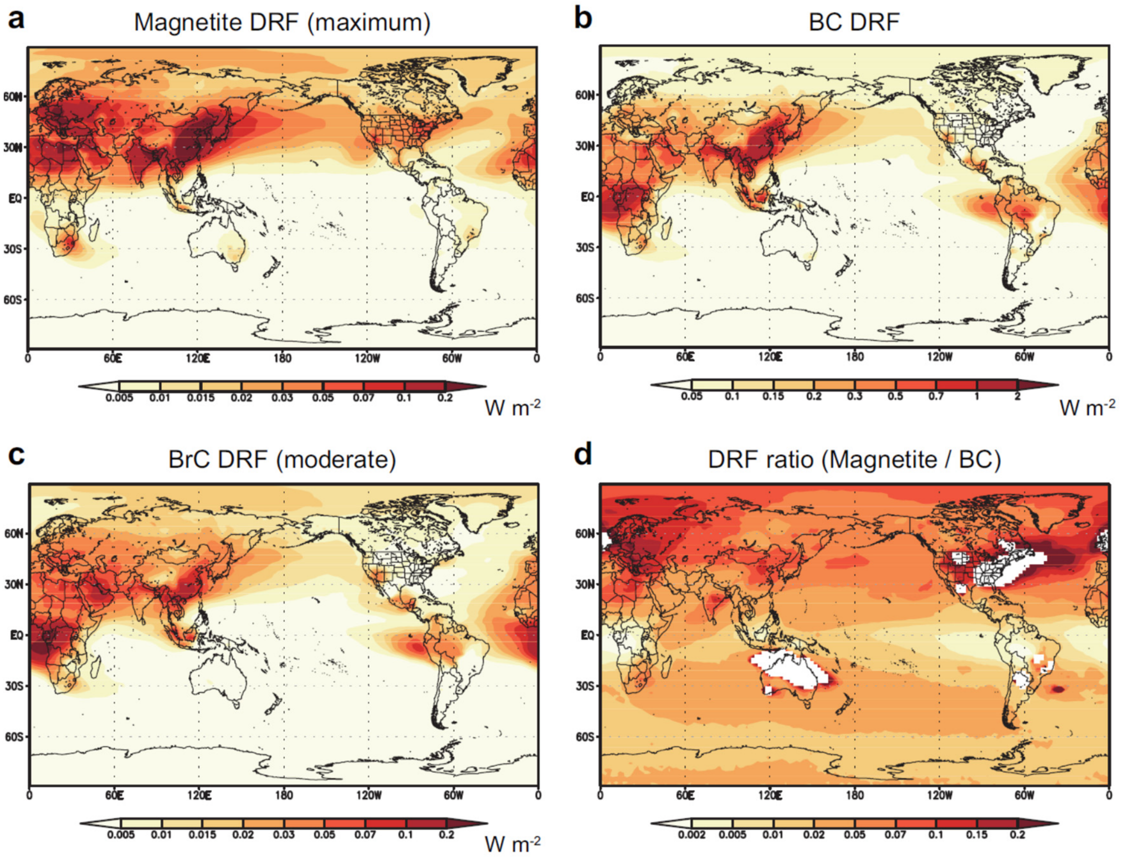

Supplementary Figure 9. Global distributions of direct radiative forcing. a-c, Direct radiative forcing (DRF) of (a) magnetite, (b) black carbon (BC), and (c) brown carbon (BrC) in the NEW simulation (5-year mean). **d,** The DRF ratio between magnetite and BC (5-year mean).

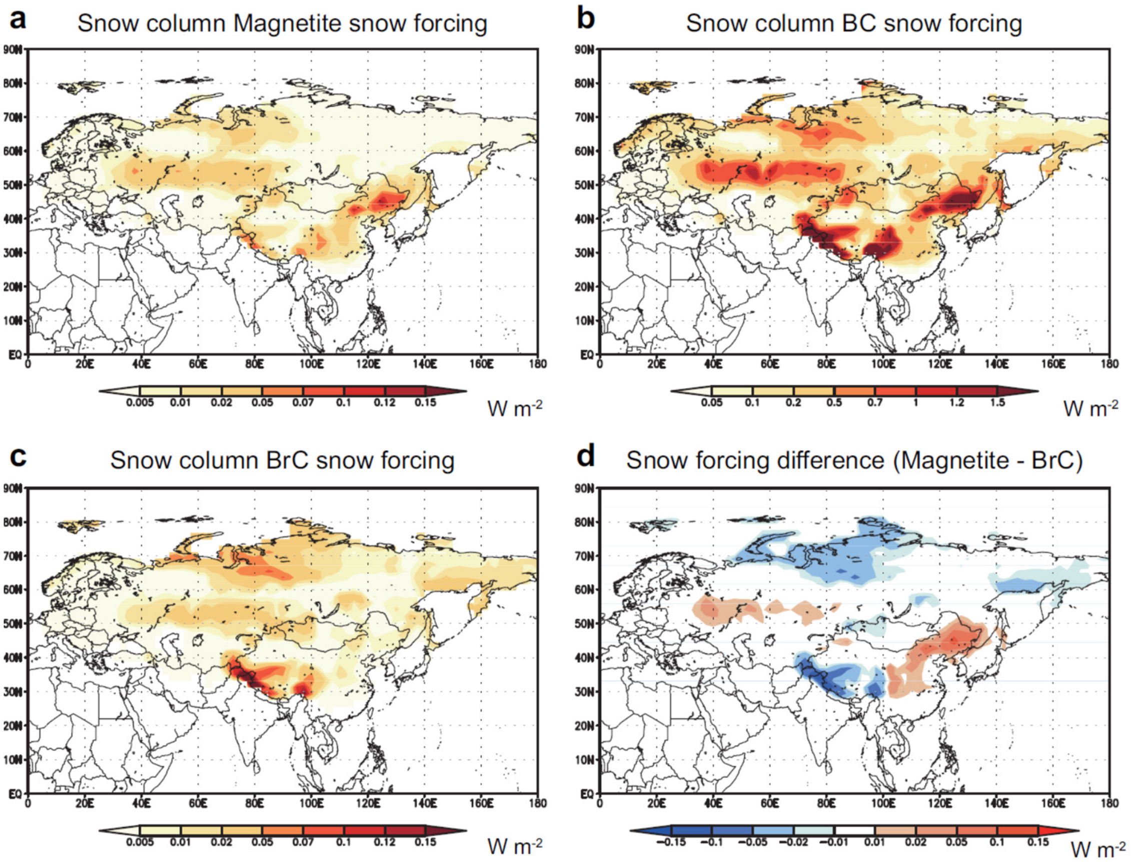

Supplementary Figure 10. Snow radiative forcing for snow column. a-c, Snow radiative forcing (see Methods) for (a) magnetite (minimum estimate), (b) black carbon (BC), and (c) BrC (moderate absorption) in the NEW simulation (5-year mean). **d,** The difference of snow radiative forcing between magnetite (minimum estimate) and BrC (moderate absorption) (5-year mean).

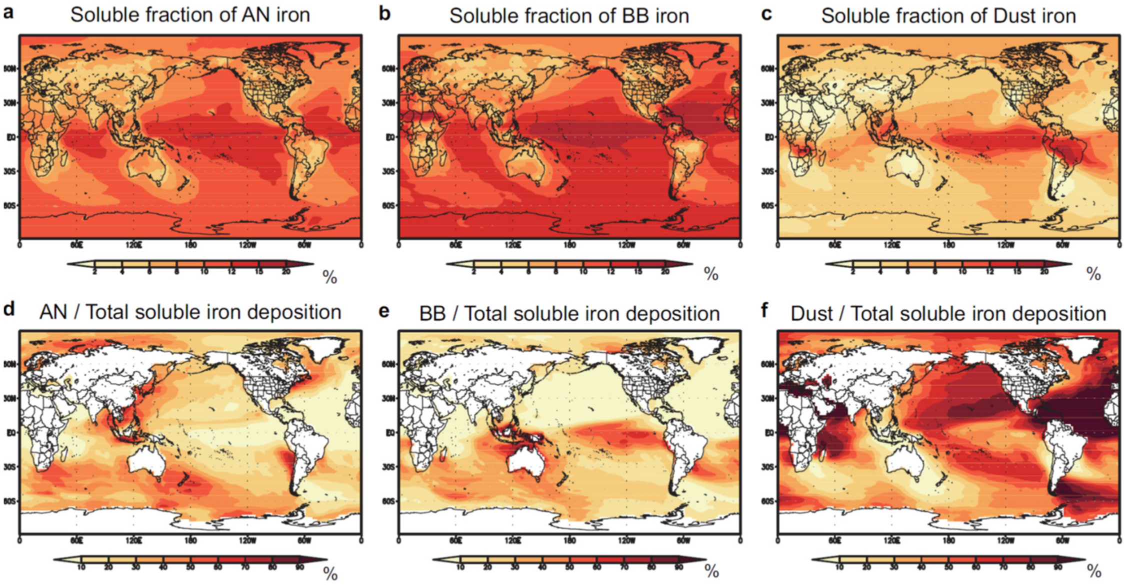

Supplementary Figure 11. Global distributions of soluble iron deposition flux. a-c, Soluble fraction (soluble/total iron deposition) for (a) combustion iron from anthropogenic (AN, fossil fuel + biofuel) sources, (b) combustion iron from biomass burning (BB) sources, and (c) iron in mineral dust (Dust) in the NEW simulation (5-year mean). **d-f,** The contribution from (d) AN, (e) BB, and (f) Dust sources to the total deposition flux of soluble iron to the ocean (AN+BB+Dust) in the NEW simulation (5-year mean).

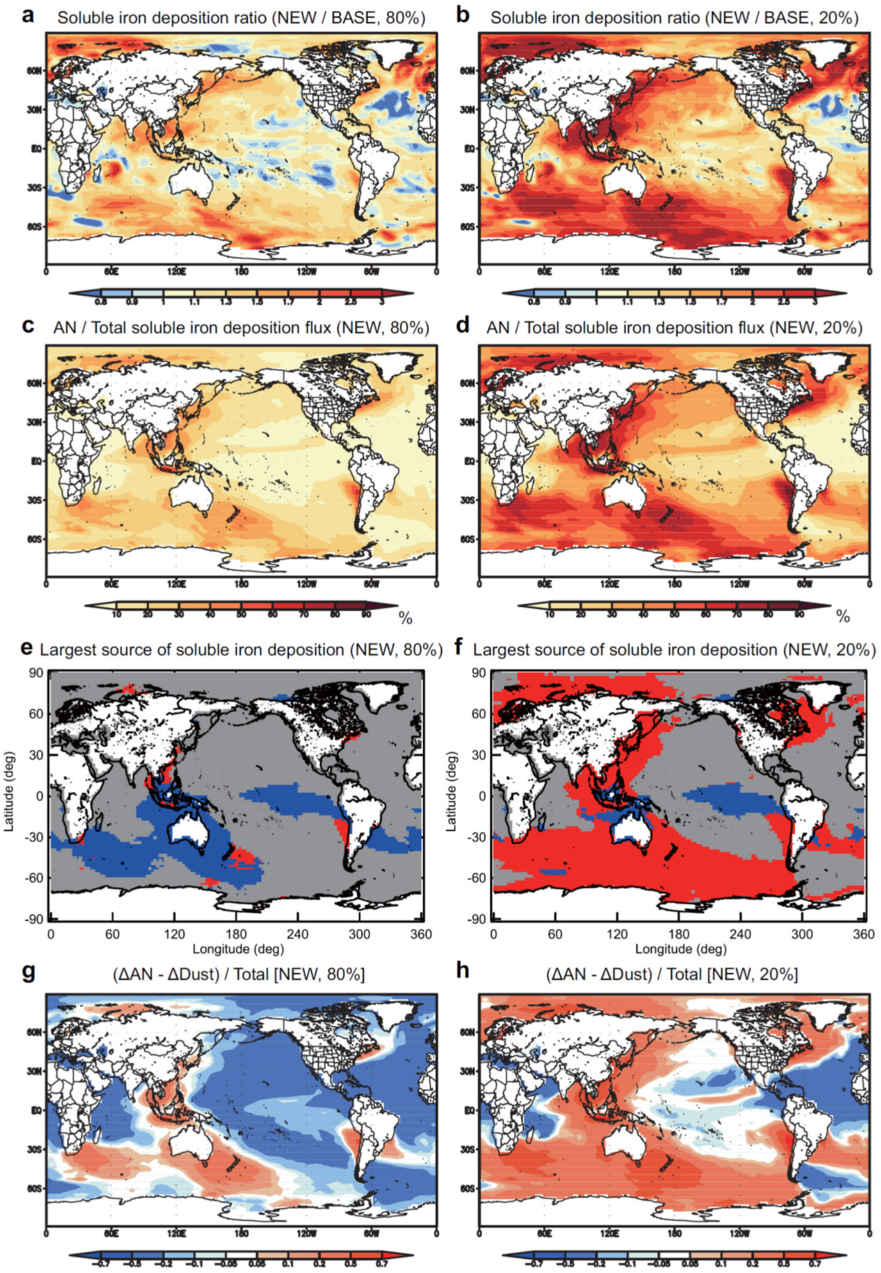

Supplementary Figure 12. Global distributions of soluble iron deposition ratio. The ratio of soluble iron deposition flux was estimated for the two assumptions: (a,c,e,f) 80% and (b,d,f,g) 20% of anthropogenic (AN) combustion iron is magnetite (5-year mean). **a-b,** Global distributions of the ratio of total soluble iron deposition flux (AN+BB+Dust) between the NEW and BASE simulations for the magnetite fraction of (a) 80% and (b) 20%. **c-d,** The contribution of AN sources to the total deposition flux of soluble iron to the ocean (AN+BB+Dust) in the NEW simulation for the magnetite fraction of (c) 80% and (d) 20%. **e-f,** The largest source of soluble iron deposition flux in the NEW simulations for the magnetite fraction of (e) 80% and (f) 20%. AN-, BB-, and Dust-dominant regions are shown by red, blue, and gray, respectively. **g-h,** Relative importance of AN and Dust sources in terms of the preindustrial to present-day changes of soluble iron deposition flux for the magnetite fraction of (g) 80% and (h) 20%. The preindustrial to present-day increases of soluble iron deposition flux were calculated for anthropogenic combustion iron (ΔAN) and iron from dust (ΔDust), and their differences were normalized by total deposition flux of soluble iron for present-day (Total) in the NEW simulation. Supplementary Figs 12e-12f were made using IGOR Pro, WaveMetrics Inc., Oswego, OR.

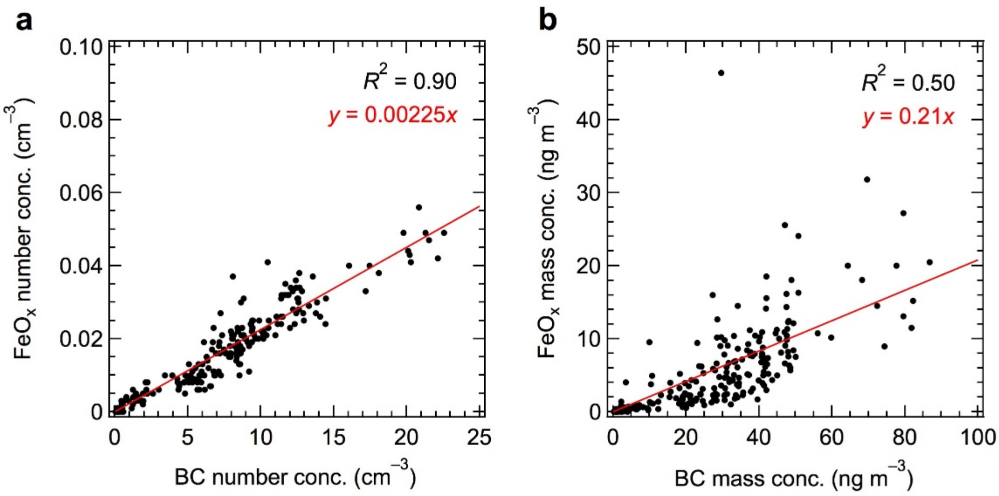

Supplementary Figure 13. Scatterplots between magnetite and BC concentrations at Zeppelin. a-b, Scatterplots of hourly observed data for (a) number and (b) mass concentrations.

Supplementary Tables

Supplementary Table 1. The treatment of magnetite and combustion iron emissions in this study

Component	Source [a]	BASE	NEW
Mass flux (PD [a])	AN	Luo et al.[1]	Magnetite/BC mass ratio in Moteki et al.[4]<br>Assumed 40% of combustion iron is magnetite
	BB	Luo et al.[1]	Luo et al.[1]
Mass flux (PI [a])	AN	10% of PD emission	10% of PD emission
Size distribution	AN	Mahowald et al.[3]	Moteki et al.[4]
	BB	Mahowald et al.[3]	Mahowald et al.[3]

[a] PD: present-day, PI: preindustrial, AN: anthropogenic (fossil fuel + biofuel), BB: biomass burning.

Supplementary Table 2. Summary of statistics in this study

Parameter	Source [a]	Unit	BASE	NEW [b]
Online global simulations (combustion iron)				
Emission flux	AN	Tg Fe y^{-1}	0.66	3.4 (1.7-6.7)
Emission flux	AN+BB	Tg Fe y^{-1}	1.7	4.4 (2.8-7.8)
Burden	AN	Tg Fe	0.0039	0.030 (0.015-0.060)
Burden	AN+BB	Tg Fe	0.014	0.041 (0.026-0.071)
Lifetime	AN	d	2.1	3.2
Heating effect of magnetite				
DRF (global)	AN	W m^{-2}	---	0.0051-0.021
DRF (East Asia [c])	AN	W m^{-2}	---	0.055-0.22
Soluble iron deposition to the ocean				
Flux (global)	AN+BB+Dust	Tg Fe y^{-1}	0.82	0.92 (0.89-1.00)
AN iron ratio (global)	---	%	1.4	8.3 (4.3-15)
Flux (30-90ºS)	AN+BB+Dust	Tg Fe y^{-1}	0.0071	0.011 (0.0087-0.015)
AN iron ratio (30-90ºS)	---	%	9.2	39 (24-56)

[a] AN: anthropogenic (fossil fuel + biofuel), BB: biomass burning, Dust: mineral dust.

[b] The values in the parentheses are the uncertainty ranges by the assumption of the fraction of magnetite to anthropogenic combustion iron (between 20% and 80%).

[c] Defined as the region within 100°-140°E and 20°-45°N.

Supplementary Note 1

Summary of uncertainties in this study

This note summarizes sources of uncertainties in our analysis, assumptions, and data shown in this study. Some of them have large uncertainties due to a lack of observational constraints. More data are necessary to reduce these uncertainties in the future.

The fraction of magnetite to anthropogenic combustion iron

One of the largest uncertainties in our analysis is the assumption of the fraction of magnetite to anthropogenic combustion iron which was assumed to be 40% in our new estimates. Magnetite was observed by a single-particle soot photometer (SP2), but no data are available for other iron-containing particles in the same measurements (total iron concentrations were evaluated by other data[1,2] as shown in Supplementary Figs 4-7). Therefore, the fraction of magnetite to anthropogenic combustion iron needs to be assumed to estimate total and soluble iron concentrations. This fraction is important because it can change our estimates of total and soluble iron concentrations from anthropogenic combustion sources by a factor of 4 within the range of the magnetite fraction between 20% and 80% (Supplementary Fig. 3).

The magnetite fraction is not well known currently. Ohata et al. (in prep) measured magnetite (observed by the SP2) and total iron concentrations (observed by an inductively coupled plasma mass spectrometry, ICP-MS) at Chiba city in Japan and showed that emissions from local steel plants had the fraction of magnetite to total iron of 25-47% for the range of mass equivalent diameter between 170 and 320 nm. Since the SP2 and ICP-MS quantify the particle size as mass equivalent diameter and aerodynamic diameter, respectively, the conversion of diameters for comparisons has some uncertainties (mainly due to the assumption of the shape factor of particles). In addition, there is no evidence that the observed fraction, which was obtained for the limited diameter range, can be extended to the whole diameter range of magnetite and combustion iron particles. However, since the magnetite fraction from steel plants is generally consistent with or slightly lower than our assumption of the magnetite fraction (40%), our assumption may be valid for industrial sources from blast furnaces. The same measurements also showed the fraction of magnetite to total iron was 42-91% in background air masses which had little influence from the local steel plants, implying that the magnetite fraction might be much greater than 40% for sources other than steel plants. Sanderson et al.[5] measured iron oxides in a roadside environment at two UK urban sites and showed the existence of FeO, Fe_3O_4 (magnetite), α-Fe_2O_3 (hematite), and γ-Fe_2O_3 (maghemite) of which γ-Fe_2O_3 was the most prominent. This result implies that magnetite may not be dominant iron oxides in transportation sources. Yu et al.[6] conducted laboratory experiments of coal combustion and showed magnetite accounted for 40-50% of total iron in terms of the mole fraction of iron in iron-containing minerals in coal ashes. Zeng et al.[7] showed the fraction of magnetite to the total iron was 14-32% in their coal combustion samples. These may imply our assumption can be used for coal combustion sources. Since each emission source may have considerably different magnetite fraction as described above, we define the uncertainty range of the magnetite fraction between 20% and 80% (a factor of 2 lower and higher fractions than 40%) and discuss total and soluble iron estimates within this uncertainty range. Simultaneous

measurements of magnetite and total iron are needed to reduce the uncertainty in the fraction of magnetite to AN iron and its spatial and temporal variations.

Emissions of magnetite and anthropogenic combustion iron

The emission flux of anthropogenic combustion iron in our new estimates depends on the assumption of the magnetite fraction described above. The emission flux has an uncertainty by a factor of 4 within the range of the magnetite fraction between 20% and 80% (shown by error bars in Fig. 1).

The spatial distribution of magnetite emissions (NEW simulation) was assumed to be the same as that of anthropogenic combustion iron emissions in Luo et al.[1] (BASE simulation). We cannot evaluate directly whether this assumption is valid or not because of no magnetite data over North America and the Southern Hemisphere. We showed simulated total iron concentrations were generally consistent with measurements in Supplementary Figs 4-7. We also showed the validity of extending the magnetite results in East Asia to the Southern Hemisphere indirectly through the estimation of possible emission sources of magnetite and the underestimation of combustion iron around Southern Africa and Australia (Supplementary Note 2). However, more data on magnetite and combustion iron are needed, especially within Southern Africa and Australia and their outflow regions. Data collection should ideally be focused on constraining uncertainties in soluble iron deposition flux to southern oceans with respect to its source contributions (AN or BB; Supplementary Note 2) in order to quantify the impact of AN activity on biogeochemistry and CO_2 uptake over the southern oceans.

Solubility of iron

Since the emissions and atmospheric processes of soluble iron are not known well, their estimates have large uncertainties. The source contributions of soluble iron (mineral dust vs. combustion iron from anthropogenic and biomass burning sources) also have large uncertainties. These are problems not only in this study but in other studies on soluble iron deposition to the ocean.

Our iron solubility estimates are derived from the global aerosol model of Scanza[8] which considers direct emissions and atmospheric formation processes of soluble iron explicitly. In this model, anthropogenic combustion iron was assumed to have the size distributions of Luo et al.[1] at emission. However, magnetite has smaller particle size distributions than combustion iron considered in Luo et al.[1] and previous studies. Since measurements suggest smaller particles have higher iron solubility[9], solubility of anthropogenic combustion iron may be much higher than the solubility data used in this study when we consider the size of magnetite particles properly in the estimation of iron solubility. This suggests that our soluble iron estimates from anthropogenic combustion sources may be underestimated in terms of iron solubility.

More field and laboratory experiments are needed to better understand processes which control solubility of iron and its spatial and temporal variability in the atmosphere. Numerical models which can represent these processes also have to be developed and evaluated.

Optical properties of magnetite and brown carbon

The direct radiative forcing (DRF) of magnetite has an uncertainty by a factor of 4 (0.0051-0.021 W m^{-2}) when we consider the inconsistency of black carbon DRF

between global aerosol model simulations[10,11] and observationally constraint estimates[12,13]. The DRF of brown carbon (BrC) has an uncertainty at least by a factor of 2 (0.016-0.038 W m^{-2}) by the uncertainty in the refractive index of BrC[14]. Since BrC emissions and formation processes were not calculated explicitly in this study, the burden and spatial distribution of BrC have some uncertainties (though global mean BrC burden is consistent with previous studies). Reducing these uncertainties is therefore necessary for more accurate estimates of magnetite and BrC DRF and their relative importance.

As a wavelength-dependent complex refractive index of magnetite, we have adopted the experimental data for pure magnetite provided by Huffman and Stapp[15], which was referenced in Supplementary Information of Moteki et al.[4]. To our knowledge, this is only the publicity-available source of wavelength-dependent optical constant of magnetite. At this point, however, we are not able to assess how this data represent the optical constants of combustion-induced aerosol magnetite.

The range of the refractive index (imaginary part) of BrC in Feng et al.[14] is 0.075-0.168 at 350 nm and 0.02-0.063 at 450 nm. These values were estimated to match the observed absorption cross sections in Chen and Bond[16] and Kirchstetter et al.[17]. Some studies, however, reported much lower imaginary part of the refractive index of BrC: 0.0082 at 350 nm[18], 0.009 at 450 nm[19], and 0.042 at 365 nm[20]. This suggests that the imaginary part of the refractive index of BrC may be more uncertain than the range of strongly and moderately absorbing BrC in Feng et al.[14]. BrC may have a lower DRF when these refractive indices are used in our simulations. Reducing the uncertainties in the imaginary part of the refractive index for magnetite and BrC is therefore important.

Comparisons with measurements

Comparisons of model simulations with observed magnetite (Supplementary Fig. 2) and total iron concentrations (Supplementary Figs 3 and 4) have uncertainties. Model simulations are not for specific years when measurements were conducted (see Methods). In addition, regions and periods are not exactly the same between measurements and model simulations. For example, each iron measurement over oceans (by cruises) represents a few days, whereas simulated results for comparisons are based on annual averages. As shown by Mahowald et al.[2], dust events are episodic and tend to occur on a few days each year, and these few events dominate the overall concentrations and deposition. Simulated iron concentrations will therefore be biased high over the regions where iron from dust is dominant. Due to the logistical constraints of ship-borne measurements, most available iron data have a limited time coverage over oceans. Longer-term observations are needed to better estimate iron amounts, especially within southern oceans.

Coarse magnetite particles

Magnetite particles greater than 3 μm in diameter are not considered in our new estimates because the current SP2 cannot measure these coarse magnetite particles[4]. In addition, the size distribution obtained by the SP2 underestimates the magnetite particle concentrations above ~0.6 μm because of the sampling loss[4]. Therefore, the emission and deposition fluxes of anthropogenic combustion iron may be underestimated in our new estimates. Measurements of coarse magnetite particles are necessary in the future for more accurate estimates of emission and deposition fluxes of anthropogenic combustion iron.

Supplementary Note 2

Extension of magnetite results in East Asia to the Southern Hemisphere

Since observations of magnetite concentrations (or magnetite/BC) are missing in the Southern Hemisphere, it is not currently possible to show direct evidence as to whether we can extend the magnetite results in East Asia to the Southern Hemisphere (Southern Africa and Australia). However, we show the validity of the extension to the Southern Hemisphere indirectly from the following two analyses using emission inventories and total iron observations.

Potential emission sources of magnetite

We examine potential emission sources of magnetite over East Asia, Europe, Southern Africa, and Australia by using the BC emission inventory used in this study[21]. The 4 results below suggest that the extrapolation of the magnetite results in East Asia and Europe (Supplementary Fig. 2) to Southern Africa and Australia is a more reasonable assumption than applying the magnetite results to the Northern Hemisphere only.

First, magnetite and BC observations show that these two species have similar temporal variations in both East Asia[4] and Europe (Supplementary Fig. 13 at Zeppelin). Though emission sources of magnetite are not known well, this result suggests that magnetite and BC have similar emission sources (from fossil fuel combustion).

Second, observed magnetite/BC ratios in East Asia and Europe are reproduced better in the NEW simulation (Supplementary Fig. 2). This result suggests that magnetite emissions used in the NEW simulation are reasonable over East Asia and Europe.

Third, BC from fossil fuel combustion is emitted mainly from the industry and transport sectors[21]. In East Asia (100°-140°E and 20°-45°N), industry sector is dominant (industry and transport sectors account for 78% and 18% of total BC emissions from fossil fuel combustion, respectively). In Europe (0°-60°E and 35°-70°N), transport sector is dominant (industry and transport sectors account for 31% and 62% of total BC emissions from fossil fuel combustion, respectively). These results suggest that industry and transport sectors may be main sources of magnetite and that the new magnetite emissions used in the NEW simulation are suitable for both the industry and transport sectors.

Fourth, BC emissions from industry and transport sectors are comparable over Southern Africa (0°-60°E and 0°-40°S, 44% from industry and 42% from transport) and Australia (110°-180°E and 10°-50°S, 37% from industry and 46% from transport). The two sectors account for 87% and 84% of total BC emissions from fossil fuel combustion over Southern Africa and Australia, respectively. These results suggest the extension of the results in East Asia (industry sector dominant) and Europe (transport sector dominant) to Southern Africa and Australia is a reasonable assumption.

Underestimation of combustion iron over Southern Africa and Australia

We compare model simulations with BC and total iron observations in the Southern Hemisphere. The 4 results below suggest that combustion iron (AN or BB sources or both) over Southern Africa and Australia and their marine outflow regions is underestimated in the model simulations and that the agreement with the iron observations is improved by extending the magnetite results in East Asia to the Southern

Hemisphere (NEW simulation).

First, BC concentrations near the surface are well simulated (or slightly overestimated) in the Southern Hemisphere in our model, as shown by Figs 9g-9h of Matsui and Mahowald[22]. This result suggests that BC emissions and concentrations are valid in the Southern Hemisphere. In contrast, surface iron concentrations and its deposition flux are underestimated around Southern Africa and Australia (Supplementary Figs 6-7). The iron/BC emissions ratio is therefore likely underestimated within these Southern Hemisphere regions.

Second, the contribution of mineral dust to total iron concentrations is low (<50%) around Southern Africa and Australia (Supplementary Figs 7c-7d). This result suggests that combustion iron emissions (AN and/or BB) are underestimated within these regions.

Third, simulated iron concentrations are comparable to observations around Amazon where the BB contribution is high (Supplementary Fig. 7e), suggesting the following two possibilities. One is the underestimation of the iron/BC ratio in AN emissions within Southern Africa and Australia. The other is the underestimation of the iron/BC ratio in BB emissions within Southern Africa and Australia because the iron/BC ratio within these regions may not be the same as (higher than) that over Amazon (the iron/BC ratio in BB emissions is assumed to be the same over Amazon and over Southern Africa and Australia in our simulations). One of these two possibilities or both contribute to the underestimation of combustion iron around Southern Africa and Australia.

Fourth, the NEW simulation has better agreement with the observations of iron concentrations and deposition flux than the BASE simulation around Southern Africa and Australia (Supplementary Fig. 7e). This suggests AN iron emissions in the NEW simulation are more realistic than those in the BASE simulation.

More magnetite and combustion iron observational data are needed in the Southern Hemisphere, particularly downwind of Southern Africa and Australia. Furthermore, attribution of combustion iron to AN and BB sources is needed to quantify the relative contribution of these sources. However, the results shown above suggest that the underestimation of AN iron emissions is a main source of the underestimation of combustion iron concentrations around Southern Africa and Australia.

Supplementary References

1. Luo, C. *et al.* Combustion iron distribution and deposition. *Global Biogeochem. Cycles*. **22**, GB1012 (2008).
2. Mahowald, N. M. *et al.* Atmospheric iron deposition: Global distribution, variability, and human perturbations. *Annu. Rev. Mar. Sci.* **1**, 245-278 (2009).
3. Mahowald, N. M. *et al.* Understanding the 30-year Barbados desert dust record. *J. Geophys. Res.* **107**(D21), 4561 (2002).
4. Moteki, N. *et al.* Anthropogenic iron oxide aerosols enhance atmospheric heating. *Nat. Commun.* **8**, 15329 (2017).
5. Sanderson, P. *et al.* Characterisation of iron-rich atmospheric submicrometre particles in the roadside environment. *Atmos. Environ.* **140**, 167-175 (2016).
6. Yu, D. *et al.* Iron transformation and ash fusibility during coal combustion in air and O_2/CO_2 medium. *Energy Fuels* **26**, 3150-3155 (2011).
7. Zeng, T., Helble, J. J., Bool, L. E. & Sarofim, A. F. Iron transformations during combustion of Pittsburgh no. 8 coal. *Fuel* **88**, 566-572 (2009).
8. Scanza, R. A. The impact of resolving mineralogy of dust on climate and biogeochemistry. PhD thesis, Cornell University (2016).
9. Baker, A. R. & Jickells, T. D. Mineral particle size as a control on aerosol iron solubility. *Geophys. Res. Lett.* **33**, L17608 (2006).
10. Myhre, G. *et al.* Radiative forcing of the direct aerosol effect from AeroCom Phase II simulations. *Atmos. Chem. Phys.* **13**, 1853-1877 (2013).
11. Samset, B. H. *et al.* Modelled black carbon radiative forcing and atmospheric lifetime in AeroCom Phase II constrained by aircraft observations. *Atmos. Chem. Phys.* **14**, 12465-12477 (2014).
12. Bond, T. C. *et al.* Bounding the role of black carbon in the climate system: A scientific assessment. *J. Geophys. Res. Atmos.* **118**, 5380-5552 (2013).
13. Myhre, G. *et al.* Climate Change 2013: The Physical Science Basis (ed. Stocker, T. F.) 659-740 (IPCC, Cambridge Univ. Press, 2013).
14. Feng, Y., Ramanathan, V. & Kotamarthi, V. R. Brown carbon: a significant atmospheric absorber of solar radiation? *Atmos. Chem. Phys.* **13**, 8607-8621 (2013).
15. Huffman, D. R. & Stapp, J. L. *Interstellar Dust and Related Topics*, J. M. Greenberg and H. C. Van de Hulst, eds. Reidel, Boston (1973), pp. 297–301.
16. Chen, Y. & Bond, T. C. Light absorption by organic carbon from wood combustion. *Atmos. Chem. Phys.* **10**, 1773-1787 (2010).
17. Kirchestetter, T. W., Novakov, T. & Hobbs. P. V. Evidence that the spectral dependence of light absorption by aerosols is affected by organic carbon. *J. Geophys. Res.* **109**, D21208 (2004).
18. Lack, D. A. *et al.* Brown carbon and internal mixing in biomass burning particles. *Proc. Natl. Acad. Sci. USA*. **109**, 14802-14807 (2012).
19. Liu, J. *et al.* Size-resolved measurements of brown carbon in water and methanol extracts and estimates of their contribution to ambient fine-particle light absorption. *Atmos. Chem. Phys.* **13**, 12389-12404 (2013).
20. Shamjad, P. M., Tripathi, S. N., Thamban, N. M., & Vreeland, H. Refractive Index and Absorption Attribution of Highly Absorbing Brown Carbon Aerosols from an Urban Indian City-Kanpur. *Sci. Rep.* **6**, 37735 (2016).
21. Lamarque, J.-F. *et al.* Historical (1850-2000) gridded anthropogenic and biomass burning emissions of reactive gases and aerosols: methodology and application. *Atmos. Chem. Phys.* **10**, 7017-7039 (2010).
22. Matsui, H. & Mahowald, N. Development of a global aerosol model using a two-dimensional sectional method: 2. Evaluation and sensitivity simulations. *J. Adv. Model. Earth Syst.* **9**, 1887-1920 (2017).
